# Sensitivity of Visual System in 5-Day “Dry” Immersion With High-Frequency Electromyostimulation

**DOI:** 10.3389/fncir.2021.702792

**Published:** 2021-12-24

**Authors:** Irina Shoshina, Inna Zelenskaya, Valeriia Karpinskaia, Yuri Shilov, Elena Tomilovskaya

**Affiliations:** ^1^Laboratory of Physiology of Vision, Pavlov Institute of Physiology, Russian Academy of Sciences, Saint-Petersburg, Russia; ^2^Institute of Biomedical Problems, Russian Academy of Sciences, Moscow, Russia; ^3^N.P. Bechtereva Institute of the Human Brain (RAS), Saint Petersburg, Russia; ^4^Department of Psychology, Samara University, Samara, Russia

**Keywords:** contrast sensitivity, “dry” immersion, gravity, electromyostimulation, illusions

## Abstract

The aim of this work was to study the sensitivity of the visual system in 5-day “dry” immersion with a course of high-frequency electromyostimulation (HFEMS) and without it. “Dry” immersion (DI) is one of the most effective models of microgravity. DI reproduces three basic effects of weightlessness: physical inactivity, support withdrawal and elimination of the vertical vascular gradient. The “dry” immersion included in the use of special waterproof and highly elastic fabric on of immersion in a liquid similar in density to the tissues of the human body. The sensitivity of the visual system was assessed by measuring contrast sensitivity and magnitude of the Müller-Lyer illusion. The visual contrast sensitivity was measured in the spatial frequency range from 0.4 to 10.0 cycles/degree. The strength of visual illusion was assessed by means of motor response using “tracking.” Measurements were carried out before the start of immersion, on the 1st, 3rd, 5th days of DI, and after its completion. Under conditions of “dry” immersion without HFEMS, upon the transition from gravity to microgravity conditions (BG and DI1) we observed significant differences in contrast sensitivity in the low spatial frequency range, whereas in the experiment with HFEMS—in the medium spatial frequency range. In the experiment without HFEMS, the Müller-Lyer illusion in microgravity conditions was absent, while in the experiment using HFEMS it was significantly above zero at all stages. Thus, we obtained only limited evidence in favor of the hypothesis of a possible compensating effect of HFEMS on changes in visual sensitivity upon the transition from gravity to microgravity conditions and vice versa. The study is a pilot and requires further research on the effect of HFEMS on visual sensitivity.

## Introduction

Understanding how the brain adapts to space flight conditions is essential for missions planning. More than half of cosmonauts returning from long flights have structural and/or functional changes in the brain accompanied by a decrease in sensorimotor characteristics and visual acuity (McIntyre and Lipshits, 2008; Grabherr and Mast, 2010; Basner et al., 2021). The ophthalmic problems developing in cosmonauts are called neuroocular syndrome associated with space flight (Laurie et al., 2019; Marshall-Goebel et al., 2019; Roberts et al., 2020; Stahn et al., 2020; Basner et al., 2021). One of the main factors affecting its development is microgravity. A decrease in gravity leads to a distortion of the gravitational vertical used by the brain to build a frame of reference and orientation in space, static and dynamic illusions, errors in assessment of the location of an object and manipulations with it (delays in the visual capture of operatively significant targets), and tracking a moving object.

“Dry” immersion (DI) is one of the most effective models of microgravity. DI reproduces three basic effects of weightlessness: physical inactivity, support withdrawal and elimination of the vertical vascular gradient (Tomilovskaya et al., 2019). The method of “dry” immersion was developed at the Institute of Biomedical Problems (Russia) in 1970s (Shulzhenko, 1975; Shulzhenko and Vill-Villiams, 1975, 1976). The main advantage of “dry” immersion included in the use of special waterproof and highly elastic fabric on of immersion in a liquid similar in density to the tissues of the human body.

The results of psychophysical studies with registration of the contrast sensitivity of the visual system using the model of “dry” immersion (DI) for simulation of the physiological effects of microgravity (Tomilovskaya et al., 2019) indicate a restructuring of the interaction of the magno- and parvocellular neuronal systems (Shoshina I. I. et al., 2020) which form, respectively, the dorsal and ventral pathways of information from the occipital to the frontal lobes of the cerebral cortex (Merigan and Maunsell, 1993; Nassi and Callaway, 2009). The interaction of these systems ensures the integrity of perception (Milner, 2017). Changing gravity conditions (gravity-microgravity-gravity) have been found to lead to an increase in contrast sensitivity in the range of low spatial frequencies (Shoshina I. I. et al., 2020), for the perception of which the magnocellular system is mainly responsible. This suggests that the magnocellular system plays a role in the processes of adaptation to changing environmental conditions; thus, it may be possible to use an assessment of its state as a marker of adaptation, which is important in preparing flight participants to a change in gravitational conditions when landing on the Moon or other objects.

The characteristics of neurons in the magno- and parvocellular systems determine the specific features of information perception (spatial-frequency filtering at the input) and high-level cognitive processes, in particular the tendency to visual illusions (Shoshina and Shelepin, 2016). According to the hypothesis of Milner and Goodale (1995) and Foley (2019), the strength of illusions decreases when the dorsal pathway is activated during the execution of a motor task, such as gripping. Based on the data on an increase in the visual contrast sensitivity in the range of low spatial frequencies and, accordingly, on the activation of the predominantly dorsal pathway upon a change in gravity conditions, we can assume the following. Upon the transition to microgravity conditions and vice versa, there will be a change in the strength of those illusions, the mechanisms of emergence of which are associated with filtering of low spatial frequencies, for example, the Müller-Lyer illusion (Ginsburg and Evans, 1979).

Understanding the nature of the observed changes, developing methods for their compensation and criteria for selecting mission participants, appropriate pre-flight preparation will minimize the risks associated with illusions of perception and spatial disorientation in microgravity conditions.

High-frequency electromyostimulation (HFEMS) is one of the means of ensuring the safety of the speed-strength qualities of muscles and work capacity of cosmonauts (Koryak et al., 2015; Kozlovskaya et al., 2016). Its effect on the motor system has been well studied, while its effects on other body systems have been studied to a lesser extent. The aim of this work was to study the sensitivity of the visual system in 5-day “dry” immersion with a course of HFEMS and without HFEMS. The hypothesis of the current study was that the use of HFEMS may be able to compensate for the effects of microgravity on visual sensitivity.

## Materials and Methods

### Participants

In this article, we present data from two studies. The first study is the dry immersion with no additional influences; the data from this study serve as a control. The second study is the “dry” immersion with HFEMS; the data from this study are presented as experimental. Each study involved 6 subjects. The average age of subjects in DI without additional influences (control) was 33.0 ± 1.5 years. In the study with HFEMS, it was 35.5 ± 1.7 years. The studies were carried out under the same conditions.

All volunteers were right-handed according to the Edinburgh Handedness Inventory (Oldfield, 1971). All studies were non-invasive and did not cause discomfort during the tests. The equipment complies with safety standards.

### General Methods

**FIGURE 1:**
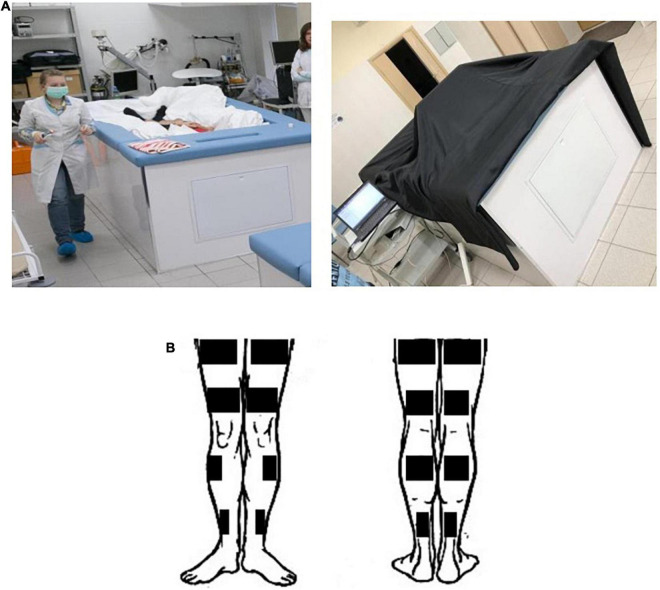
Conditions of “dry” immersion experiment. **(A)** The demonstration of conditions in the DI experiment to assess the contrast sensitivity. **(B)** A scheme of electrode positioning for the HFEMS.

Each volunteer was in DI conditions for 5 days: he was immersed in a bath 200 × 100 × 100 cm in size filled with water, the temperature of which was maintained at 33.0 ± 0.5°C. The surface of the water was covered with a freely floating waterproof fabric, the area of which was more than 2 times the area of the water surface. Thus, the test subject while being immersed in the water was isolated from direct contact with it (Figure 1A).

A test subject was immersed into a deep bath up to the neck level, in a supine position. The folds of the waterproof fabric allowed the person’s body to be enveloped from all sides freely. The high elasticity properties of the fabric artificially created conditions similar to zero gravity via flotation. The detailed description of the Dry Immersion model and its effects on the human body can be found in the review (Tomilovskaya et al., 2019).

The daily routine was specified in accordance with the schedule of studies, including 8 h of sleep, 3–4 meals, a medical supervision program and experimental studies. The research participants were taken out of DI for 15–20 min each day for sanitary and hygienic procedures (mostly in supine position) with the usage of a special lift rising from the bottom of the bath. The subjects keyed almost immobile: they had the opportunity to move, but they had the strong recommendations to limit their motor activity, and that was controlled by the researchers. The visual environment for the participants was common - the light from windows and from artificial lights, darkness during the night. The subjects could also use their computers and smartphones, to read the books during their free time in the course of exposure.

Testing procedure was as follows: 24 h before the start of DI (background—BG), then on the 1st, 3rd, and 5th days of DI (DI1, DI3 and DI5, respectively) and 5 h after the completion of DI (DI5 + 5h). According to the data of previous studies, the restoration of sensorimotor functions occurs within the first 24 h after the completion of DI, therefore the last study was carried out 5 h after the completion of the immersion. The BG stage of the study was carried out in a filled bath, in a supine position (Sosnina et al., 2021). The study in DI was carried out in the morning, after breakfast and all necessary hygienic procedures.

### High-Frequency Electromyostimulation

HFEMS was performed using an Amplidin-EST stimulator which is a source of alternating sinusoidal current with a carrier frequency of 2,000 Hz, interrupted by rectangular pulses with a frequency of 50 Hz (full modulation amplitude) and a duration of 10 s. Stimulation training of the quadriceps femoris muscle (QFM), triceps surae muscle (TSM) and tibialis anterior muscle (TAM) was carried out on two limbs of the subject by the direct bipolar electrical stimulation of the muscles according to the method of Kots and Khvilon (1971) and Koryak (2018), but in isotonic mode of muscle contractions in supine position. The angle in the knee joint was 180°, in the ankle joint—about 130°. Physiotherapeutic conductive rectangular electrodes (40 × 100 mm—for TAM, 45 × 200 mm and 40 × 160 mm—for QFM, and 40 × 160 mm and 40 × 100 mm—for TSM) were placed over the entire width of the stimulated muscle (Figure 1B). Each subject was familiarized with the HFEMS procedure before the start of the DI. Familiarization procedure was exactly the same as the experimental sessions. During familiarization sessions the comfortable intensity of stimuli for each muscle was defined. HFEMS was performed by applying rectangular rhythmic pulses at a frequency of 50 Hz; in addition, each pulse, the duration of which was 10 ms, was “filled” with a carrier sound frequency of 2,500 Hz. The stimulation amplitude ranged from 4.3 to 19.0 V. The subjects got the HFEMS in the course of DI, but not inside the bath. For safety reasons, the stimulation was performed outside the immersion bath, on a half-deflated air mattress to maintain support unloading conditions. The stimulation procedures were carried out every day in DI for 20 min a day [10 stimulations × (10 s + 50 s) = 10 min].

The sensitivity of the visual system was assessed by measuring contrast sensitivity and magnitude of the Müller-Lyer illusion.

### Visual Contrast Task

The contrast sensitivity of the visual system was registered in the ranges of low, medium, and high spatial frequencies. The neurons of the magnocellular system are more specific to the perception of low spatial frequencies, while the neurons of the parvocellular system—to high spatial frequencies (Merigan and Maunsell, 1993; Nassi and Callaway, 2009). Since the medium spatial frequencies are processed by the neurons of both systems, we considered the contrast sensitivity in this range of spatial frequencies as a measure of the incongruity of their interaction.

**FIGURE 2:**
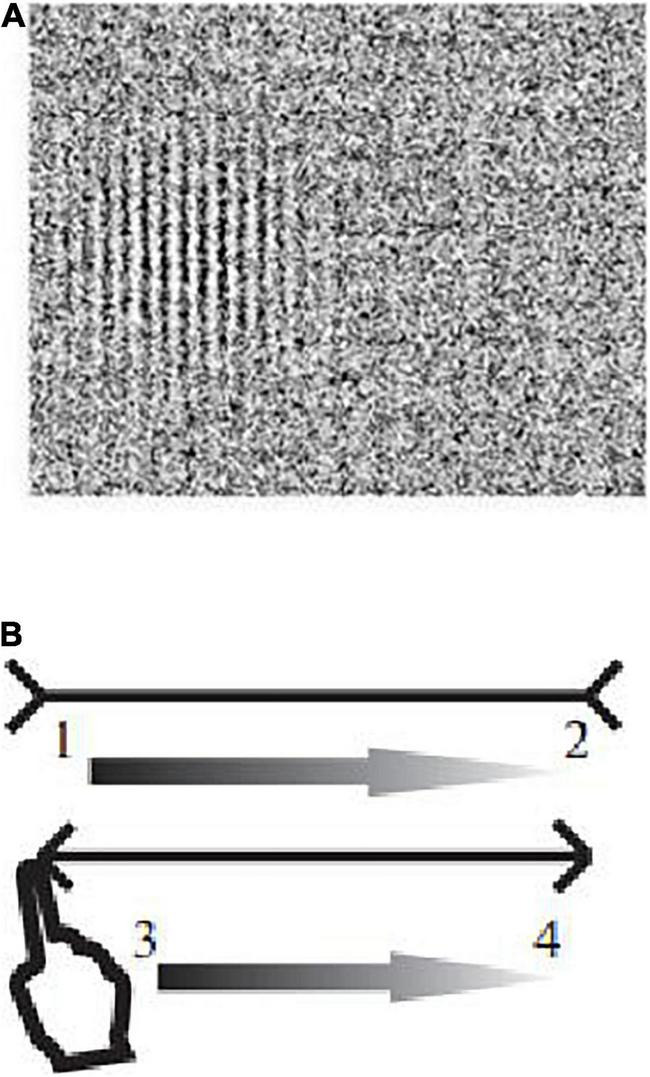
Examples of images in the DI experiment. **(A)** Examples of images of Gabor elements presented in the study to assess the contrast sensitivity in different ranges of spatial frequencies. **(B)** The Müller-Lyer illusion (1, 2, 3, 4—the direction of movement of the finger along the segments of the Müller-Lyer figure).

The contrast sensitivity was registered upon presentation of Gabor elements with spatial frequencies of 0.4, 0.8, 1.0, 3.0, 6.0, and 10.0 cycles/degree on the screen of a Toshiba Satellite A200-1M8 monitor (Intel^®^ Core2™ Duo Processor T7100 1.8 GHz/1 GB, 15.4” display, TFT WXGA high brightness active matrix (Toshiba TruBrite), 1,024 × 600 pixel resolution, 60 Hz refresh rate). When analyzing the data, the frequencies of 0.4 and 0.8 cycles/degree were attributed to the range of low spatial frequencies, 1.0 and 3.0 cycles/degree—to the range of medium frequencies, 6.0 and 10.0 cycles/degree—to the range of high frequencies. Stimuli were displayed in random order to the left or to the right of the center of the screen (Figure 2A). The subject’s task was to click on the right mouse button if he sees an image on the right, and the left button, if on the left. The choice had to be made even when the subject was not sure if he saw the test image.

To present the Gabor elements and assess the contrast sensitivity, we used a computer program that makes it possible to create test images on a monitor of any type without preliminary calibration. To render the brightness profile of the test images, it uses variations in the density of white points randomly positioned on a black background rather than shades of gray color.

The duration of the stimulus was not limited. Threshold contrast measurement is implemented in the program using the adaptive staircase procedure. The presentation of stimuli was started with a contrast of 0.5. If the subject thrice gave the correct answer in which half of the screen the image was displayed, the program reduced the contrast by 20%. If even one mistake was made, the contrast was increased by 20%. Then this cycle was repeated with a contrast change increment of 20%. As a result, the contrast of the test image gradually decreased to the threshold level at which the probability of making a mistake was 0.5. The measurements were ended when the contrast value made a predetermined number of such oscillations—“reversals.” The number of reversals for each test spatial frequency was 8. The threshold was calculated programmatically as the average value of contrasts at the points of “reversals.” The threshold was taken as the contrast value at which the subject gave the correct answer with a probability of *P* = 0.794 (since the probability of three correct answers in a row is P × P × P = 0.5, then $P={}^{3}\sqrt{0.5}$ ≈ 0.794).

To standardize the conditions for the observation of the stimuli, the immersion bath with a monitor mounted on it was covered with a dense black cloth that did not allow sunlight to pass through (Figure 1). The monitor was positioned at a distance of 1.5 m from the subject so that his eyes were directed approximately into the center of the screen. The test subject was in a reclining position with a pillow under head. The observation was binocular. The visual acuity of all volunteers was within the normal range.

### Müller-Lyer Illusion Task

The strength of the Müller-Lyer illusion was determined by presenting two types of images, each of which contained two horizontal segments that the subject had to compare with each other. The first type of images consisted of “neutral” segments of equal length that did not cause illusions (control stimulus). In the second type of images, the upper segment was framed by “arrow tails,” and the lower one—by “arrow points”—a variant of the Müller-Lyer illusion (Figure 2B). For each type of stimulus, segments of five lengths were used: 4.0, 5.5, 7.0, 8.5, and 10.0 cm. The lengths of segments in a pair were changed from trial to trial in random order. The task of the subject consisted in the sensorimotor assessment of the lengths of the segments with the leading hand.

When an image was presented, the test subject moved the index finger of the leading hand from left to right along the upper and lower central segments (first along the upper segment, then along the lower segment) that he saw in front of him. We used 6 images: 2 types of stimuli (neutral segments, Müller-Lyer illusion) with 3 pairs of segments each. For each type of stimulus, the program randomly selected 3 pairs of segments of different lengths from the above set of lengths (both segments in a pair had the same length, but the lengths of pairs of segments between individual trials were different). First, three pairs of neutral segments were presented, then three pairs of segments framed by arrow points causing the Müller-Lyer illusion.

The stimuli in this study were presented on an Iiyama Prolite T2252MTS touchscreen monitor (Japan) with the size of the visible area of 476.4 × 268.11 mm, resolution—1,920 × 800 pixels, γ—2.2, white color temperature—6,500 K, illumination during touching—200 cd/m^2^. The reclining subject with a pillow under head was positioned 60 cm from the monitor screen. On the touchscreen monitor, the program determined the coordinates of the pixels in which the subject touched the leftmost and rightmost points of the central segments, i.e., the starting and ending points of hand movements along the segments. On the basis of these coordinates, the lengths of the segments were calculated as the Euclidean distance between the starting and ending points of the hand movement; the relative strength of illusions was calculated as the difference in the lengths of the segments indicated by the subject divided by the real length of the segments. The strength of the illusion was considered positive if the subject overestimated the upper segment, and negative if he overestimated the lower segment.

### Methods of Statistical Analysis

All data are presented as mean ± SEM. The significance of differences in contrast sensitivity was assessed using the two-way ANOVA, mixed ANOVA and non-parametric Mann-Whitney test. The two-way ANOVA was used to analyze the effects of different factors in experimental conditions: Day (BG, DI1, DI3, DI5, DI5 + 5h) and Freq (Low, Medium, High), followed by a *post hoc* comparison using LSD. Statistical analysis using the mixed ANOVA included factors: Day (BG, DI1, DI3, DI5, DI5 + 5h), Freq (Low, Medium, High) and Group [Experimental (with HFEMS)/Control (without HFEMS)], followed by a *post hoc* comparison using LSD. The significance of differences in the strength of illusion was assessed using the mixed ANOVA. Statistical analysis was performed for the averaged data from three trials for the following factors: Stimulus [NO (neutral)/ML (Müller-Lyer illusion), Day (BG, DI1, DI3, DI5, DI5 + 5h) and Group (Experimental/Control)]. *P* < 0.05 was considered statistically significant. Data analysis was carried out with the SPSS Inc. software.

## Results

**FIGURE 3:**
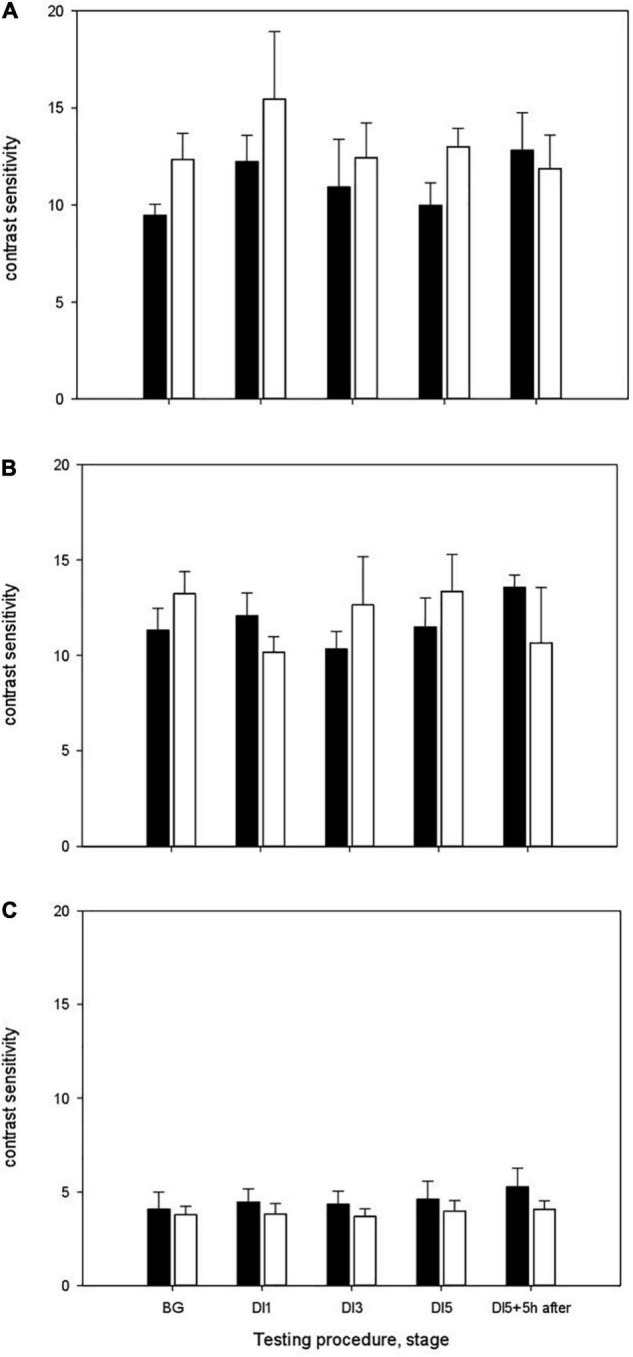
The contrast sensitivity of the visual system in the DI with HFEMS (white bars) and without HFEMS (black bars). **(A)** The contrast sensitivity in the range of low spatial frequencies. **(B)** The contrast sensitivity in the range of medium frequencies. **(C)** The contrast sensitivity in the range of high frequencies. The ordinate shows contrast sensitivity, the inverse of the contrast threshold. All data are presented as mean ± standard error.

The results of measurements of contrast sensitivity in different ranges of spatial frequencies are shown in Figure 3. During study without using the HFEMS, the background (BG) values of contrast sensitivity in the range of low spatial frequencies were 9.46 ± 0.57, medium frequencies –11.32 ± 1.13, high frequencies–4.09 ± 0.90 (Figure 3A). On the 1st day of exposure to DI (DI1), the contrast sensitivity was in the range of low spatial frequencies 12.23 ± 1.36, medium—12.06 ± 1.19, high frequencies–4.45 ± 0.70. On the 3rd day of DI (DI3), the contrast sensitivity in the range of low spatial frequencies was 10.92 ± 2.45, medium frequencies–10.3 ± 0.92, high frequencies–4.34 ± 0.70. On the 5th day of DI (DI5), the contrast sensitivity was 9.97 ± 1.16, 11.48 ± 1.52, and 4.60 ± 0.97, respectively. After the completion of immersion, the contrast sensitivity in the range of low spatial frequencies was 12.82 ± 1.92, medium–13.57 ± 0.63 and in the range high frequencies–5.27 ± 0.99.

Statistical analysis using the two-way ANOVA (Repeated Measures) for DI without HFEMS showed that the main influence was exerted by the factors Day (stage) [*F*(4, 20) = 4.73, *p* = 0.008, η^2^ = 0.486] and Spatial Frequency [*F*(2, 10) = 40, *p* < 0.001, η^2^ = 0.889]. Pairwise Comparisons showed a significant increase in contrast sensitivity in the range of low spatial frequencies upon the transition from gravity to microgravity conditions (between BG and DI1, *p* = 0.03). The differences in contrast sensitivity between BG and DI5 + 5 h were at the tendency level (*p* = 0.08), as well as between DI5 and DI5 + 5 h (*p* = 0.08). Statistical analysis using the Mann-Whitney test confirmed the significance of differences in contrast sensitivity in the range of low spatial frequencies upon the transition from gravity to microgravity conditions (*Z* = –2.01, *p* = 0.03, df = 10), the tendency to differences between BG and DI5 + 5 h (*Z* = –1.76, *p* = 0.08) and between DI5 and DI5 + 5 h after the completion of immersion (*p* = 0.08).

During study with HFEMS, the background (BG) values of contrast sensitivity in the range of low spatial frequencies were 12.35 ± 1.34, medium frequencies–13.22 ± 1.16, high frequencies—3.78 ± 0.46 (Figure 3B). On the 1st day of exposure to DI (DI1), the contrast sensitivity was in the range of low spatial frequencies: 15.45 ± 3.49, 10.16 ± 0.81 and 3.82 ± 0.55, respectively. On the 3rd day of DI (DI3), the contrast sensitivity in the range of low spatial frequencies was 12.43 ± 1.79, medium–12.64 ± 2.52, and in the range high frequencies–3.69 ± 0.40. On the 5th day of DI (DI5), the contrast sensitivity was: 12.99 ± 0.94, 13.34 ± 1.95 and 3.98 ± 0.55, respectively. Five hours after the completion of immersion (DI5 + 5 h), the contrast sensitivity in the range of low spatial frequencies was 11.87 ± 1.72, medium frequencies–10.64 ± 2.91, high frequencies–4.07 ± 0.44.

Statistical analysis of the data indicated that there are significant differences in the contrast sensitivity in the range of medium spatial frequencies between BG and DI1 (*p* = 0.001). The results of statistical analysis using the Mann-Whitney test confirmed this result (*Z* = –2.40, *p* = 0.02, df = 10). Five hours after the completion of DI, we observed no significant changes in contrast sensitivity in response to changes in gravitational conditions. Two-way ANOVA for DI with HFEMS showed that the main influence was exerted only by the factor Spatial Frequency [*F*(2, 10) = 24.5, *p* < 0.001, η^2^ = 0.83].

Thus, under the conditions of “dry” immersion with HFEMS, upon the transition from gravity to microgravity conditions (BG and DI1) significant differences in contrast sensitivity were observed in the range of medium spatial frequencies, whereas in the experiment without HFEMS—in the range of low spatial frequencies.

Statistical analysis using the mixed ANOVA showed that the main influence was exerted by the factors Spatial Frequency [*F*(2, 9) = 48.65, *p* = 0.0001, η^2^ = 0.915] and Day × Group [*F*(4, 7) = 4.64, *p* = 0.038, η^2^ = 0.726]. No significant interaction of the factors Group × Day × Spatial Frequency was found. Significant interaction of the factors Group × Day serves as evidence in favor of differences in the dynamics of changes in contrast sensitivity in the control and experimental groups. However, the lack of significant three-way interaction does not yet allow us to unequivocally assert that the groups are different.

The results of the sensorimotor assessment of the segments indicate that the subjects correctly assessed the neutral segments; the strength of overestimation of the size of the upper segment did not significantly differ from zero and was in the range of –0.9 to 1.1%.

**FIGURE 4:**
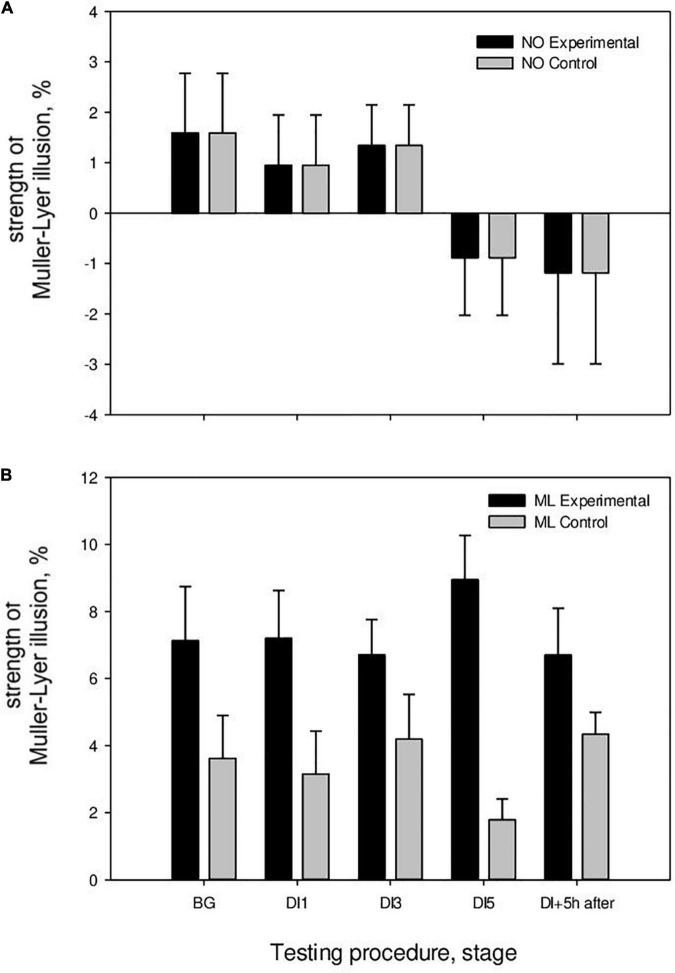
The strength of the Müller-Lyer illusion in the DI with HFEMS and without HFEMS. **(A)** The results of the perception of neutral segments. **(B)** Results of perception of segments with arrows (M ller-Lyer illusion). All data are presented as mean ± standard error.

In the study without HFEMS, the strength of the Müller-Lyer illusion in the BG stage was 3.95 ± 1.25%, on the 1st day of immersion (DI1)—3.15 ± 1.23%, on the 3rd day (DI3)—4.18 ± 1.26%, on the 5th day (DI5)—1.79 ± 0.63%, and 5 h after the completion of immersion (DI5 + 5 h)—4.34 ± 0.69% (Figure 4). A significant decrease in the strength of the Müller-Lyer illusion was registered on the 5th day of DI (*p* = 0.03), followed by an increase after the completion of DI. The strength of the Müller-Lyer illusion was above zero only after the completion of DI (*p* = 0.002). Thus, under microgravity conditions without additional influences, the Müller-Lyer illusion was absent (Table 1).

**TABLE 1:** The strength of the Müller-Lyer illusion in comparison with neutral segments.

Group	Day	(I) Stimulus	(J) Stimulus	Mean difference (I-J)	Std. Error	Sig.^a^
With HFEMS	DI1	NO	ML	–8.138*	1.954	0.000
	DI3	NO	ML	–6.575*	1.524	0.000
	DI5	NO	ML	–6.916*	1.891	0.001
Without HFEMS	DI1	NO	ML	–2.204	1.863	0.246
	DI3	NO	ML	–2.844	1.453	0.060
	DI5	NO	ML	–2.684	1.803	0.147

*Based on estimated marginal means.*

*The mean difference is significant at the 0.05 level.

*^a^Adjustment for multiple comparisons: Least Significant Difference (equivalent to no adjustments).*

During study with HFEMS, the strength of the Müller-Lyer illusion in the background stage was 7.13 ± 1.13%, on the 1st day of immersion (DI1)—7.19 ± 1.08%, on the 3rd day (DI3)—6.71 ± 0.78%, on the 5th day (DI5)–8.95 ± 1.17%, and 5 h after the completion of immersion (DI5 + 5 h)–6.70 ± 0.95% (Figure 4). The strength of the Müller-Lyer illusion was above zero on all days of measurements (Table 1).

Statistical analysis using the mixed ANOVA showed that the main influence was exerted by the following factors: (Tests of Between-Subjects Effects) Group [*F*(1, 19) = 9.67, *p* = 0.006, η^2^ = 0.337]; Stimulus [*F*(1, 19) = 46.18, *p* < 0.001, η^2^ = 0.708]; Group × Stimulus [*F*(2, 19) = 6.6, *p* = 0.019, η^2^ = 0.258]; (Multivariate Tests) Day × Group [*F*(4, 16) = 3.6, *p* = 0.028, η^2^ = 0.475]. The magnitude of the Müller-Lyer illusion differed between the control (without HFEMS) and experimental (with HFEMS) groups on DI1 (*p* = 0.04) and DI5 (*p* = 0.001).

## Discussion

The hypothesis of this study was that the use of HFEMS could possibly compensate for the negative effects of microgravity on the contrast sensitivity of the visual system and the strength of the Müller-Lyer illusion.

The contrast sensitivity of the visual system reflects the characteristic features of perception and analysis of information at the input. From the standpoint of the theory of spatial-frequency filtering, visual perception is a result of distinguishing the spatial-frequency characteristics of visual stimuli by a set of relatively “narrow” channels (Campbell and Robson, 1968). Channels are neural complexes tuned to the perception of different spatial frequencies. There are many channels; the main ones are magno- and parvocellular channels or systems. The results of numerous studies indicate that a change in the activity of one of these systems leads to an incongruity in their interaction (Shoshina et al., 2014; Shoshina and Shelepin, 2016; Shoshina et al., 2018; Shoshina I. et al., 2020; Shoshina I. I. et al., 2020; Zemon et al., 2020). The activity of the magnocellular system has been shown to increase, which leads to a shift in the balance in the interaction of two opposing systems. The importance of the coordinated work of the magno—and parvocellular systems is shown on the model of chronic stress during burnout (Shoshina et al., 2018) and psychopathology (Shoshina et al., 2014; Shoshina and Shelepin, 2016; Shoshina I. et al., 2020; Zemon et al., 2020).

The results of studies of the effect of microgravity on the contrast sensitivity of the visual system in the course of a 21-day DI indicate a change in the functional state of the magnocellular system when the gravity conditions are altered (Shoshina I. I. et al., 2020). The activity of the magnocellular system has been shown to increase with a resulting shift within the balance within the interaction of two opponent systems. The results of studies of the visual system of cosmonauts before and after long orbital flights also indicate that there are changes in the activity of the magnocellular system when the gravity conditions are altered. Danilichev et al. (2019) registered a gradual recovery (on days 1, 3, 7, and 14 after a long flight) of contrast sensitivity in the range of low spatial frequencies to the background level measured before the start of the flight. Thus, the data indicate the possibility of using the activity of the magnocellular system as a marker of the functional state, a marker of the adaptation process. The neurons of the magnocellular system send their axons mainly along the dorsal pathway to transmit information from the caudal region to the frontal cortex (Merigan and Maunsell, 1993). The studies with the registration of electroencephalograms in cosmonauts in the process of their solving navigation problems have also shown that microgravity leads to functional reorganization of the dorsal pathway (Cheron et al., 2014).

The present study of 5-day “dry” immersion with the course of HFEMS indicates that on the 1st day of DI the average values of contrast sensitivity not increase in the range of low spatial frequencies. At the same time, in DI without additional influences, there is an increase in contrast sensitivity in the range of low spatial frequencies upon a change in gravity conditions. However, in a study using myostimulation, a decrease in contrast sensitivity in the range of medium spatial frequencies was observed. Such a decrease is considered by us as evidence of an increase in the level of internal noise of the visual system. One of the factors of its increase is likely to be an incongruity in the interaction of the magno- and parvocellular systems.

The dynamics of changes in the sensorimotor assessment of illusion and neutral segments in the 5-day DI without HFEMS is qualitatively similar to the data that we obtained earlier with 21-day DI and, in which the subjects were not exposed to other influences but DI (Sosnina et al., 2021). In the 5-day DI without HFEMS, the dynamic of the change in the magnitude of the Müller-Lyer illusion indicates a decrease in the strength of the illusion during the immersion. In study with HFEMS, there was no decrease in the strength of the Müller-Lyer illusion, as in immersion without additional influences.

According to Clément et al. (2009), a decrease in the strength of illusions occurs during systemic (otolithic) dizziness associated with disturbances in the functioning of the vestibular system. In conditions of flight along a parabolic trajectory with a short period of microgravity (for only 20 s), a decrease in the strength of geometric visual illusions has also been shown (Villard et al., 2005). In our study of 5-day immersion with HFEMS, this decrease did not occur. It can be assumed that HFEMS, increasing the afferent stream under conditions of its deficit during support unloading (Koryak, 2018), normalizes the sensorimotor interaction.

We consider the data of these studies from the point of view of the theory of two pathways—dorsal and ventral (Goodale and Milner, 1992). The dorsal pathway for “action” with projections from the primary visual areas to the posterior parietal cortex plays a crucial role in real-time action control; it converts information about the location and positioning of target objects into the coordinate systems, used by the effectors to perform an action (Goodale, 2014; Foley et al., 2015). The neurons of the magnocellular system that are sensitive to the perception of low spatial frequencies, send their axons mainly toward the dorsal stream and provide the functions of perception of movement, spatial localization, visual-spatial orientation, and global analysis of the visual field (Merigan and Maunsell, 1993; Nassi and Callaway, 2009).

The ventral pathway for “perception” with projections from the primary visual areas into the inferior temporal cortex helps to create rich and detailed visual representations of the world that allow us to identify objects and events, give them meaning and significance, establish cause-and-effect relationships. The ventral pathway associated with the structures of the temporal and frontal lobes involved in memory, emotions, and social behavior (Foley et al., 2015).

The dorsal and ventral systems are involved in two different frames of reference that provide the function of spatial orientation (Baizer et al., 1993; Milner and Goodale, 1995; Klatzky, 1998; Gramann et al., 2010). Calculations required for seeing “to perceive” are very different from calculations required for seeing “to act.” The dorsal system provides an egocentric frame of reference focused on absolute values and visual-spatial orientation, the ventral system—an allocentric frame of reference focused on the structure of the scene and coding of the size, orientation and location of objects relative to each other. Owing to the coordinated work of the systems with perceptual representations based on objects and scenes, a coherent view of the environment, location and direction of navigation in it is formed (Milner, 2017). Extreme impacts (stress) lead to an incongruity in their interaction, the nature of which may differ. In particular, the change in gravity conditions is accompanied by a shift of the activity locus toward the dorsal system (Shoshina I. I. et al., 2020), which, as we assume, can be compensated for by additional afferentation using HFEMS. However, further research is required to test this assumption.

McGregor et al. (2021) investigated resting−state functional connectivity (FC) during a spaceflight analog (30 days of strict head down−tilt bed rest in elevated ambient carbon dioxide (HDBR + CO_2_). A subset of participants developed optic disc edema, a sign of spaceflight−associated neuro−ocular syndrome (SANS). Subjects who developed optic disc edema exhibited a distinct pattern of FC changes within visual and vestibular−related networks during the intervention. This finding confirms that SANS impacts not only neuro−ocular structures, but also functional brain organization.

Therefore, further prospective investigations studies, including sensory assessments, are significant to research the observed differences.

## Conclusion

Conditions of space flight lead to changes in sensorimotor characteristics that are responsible for fundamental skills necessary for piloting and landing aircraft and spacecraft, driving various vehicles, handling manipulators and other devices. As a result of a decrease in gravity (and, probably, other factors), there is a loss of a spatial reference point (support) which is perceived by the receptors of the vestibular, proprioceptive, tactile and visual systems and is used by the central nervous system for spatial orientation, navigation and coordination of movements. In this connection, of importance is not only the problem of studying changes in sensitivity under conditions of altered gravity, but also the search for ways to compensate for negative effects. Since the opportunities for research in space are limited, methods of gravitational unloading in terrestrial conditions are used. “Dry” immersion is a popular model for such gravitational unloading.

In the present study, during a 5-day “dry” immersion, we studied the contrast sensitivity of the visual system in different ranges of spatial frequency, as well as the strength of the Müller-Lyer illusion in the sensorimotor tracking task.

Under conditions of “dry” immersion without HFEMS, upon the transition from gravity to microgravity conditions (BG and DI1) we observed significant differences in contrast sensitivity in the low spatial frequency range, whereas in the experiment with HFEMS—in the medium spatial frequency range. In the experiment without HFEMS, the Müller-Lyer illusion in microgravity conditions was absent, while in the experiment using HFEMS it was significantly above zero at all stages. Thus, we obtained only limited evidence in favor of the hypothesis of a possible compensating effect of HFEMS on changes in visual sensitivity upon the transition from gravity to microgravity conditions and vice versa. The study is a pilot and requires further research on the effect of HFEMS on visual sensitivity.

## Data Availability Statement

The original contributions presented in the study are included in the article/supplementary material, further inquiries can be directed to the corresponding author/s.

## Ethics Statement

The studies involving human participants were reviewed and approved by the Bioethics Committee of the Institute of Biomedical Problems of the Russian Academy of Sciences (Moscow). The patients/participants provided their written informed consent to participate in this study.

## Author Contributions

IS wrote the manuscript, made the statistical analysis of the data, and made its revisions. IS, YS, and VK participated in conceiving designing of the study. IZ conducted the experimental research in DI. ET organized and supervised the experimental work and contributed in the global revision of the manuscript and made its revisions. All authors read and approved the current manuscript.

## Conflict of Interest

The authors declare that the research was conducted in the absence of any commercial or financial relationships that could be construed as a potential conflict of interest.

## Publisher’s Note

All claims expressed in this article are solely those of the authors and do not necessarily represent those of their affiliated organizations, or those of the publisher, the editors and the reviewers. Any product that may be evaluated in this article, or claim that may be made by its manufacturer, is not guaranteed or endorsed by the publisher.
